# Programmed death-ligand 1 (PD-L1) expression in primary gastric adenocarcinoma and matched metastases

**DOI:** 10.1007/s00432-023-05142-x

**Published:** 2023-07-25

**Authors:** Drolaiz H. W. Liu, Heike I. Grabsch, Beat Gloor, Rupert Langer, Bastian Dislich

**Affiliations:** 1 Department of Pathology, GROW School for Oncology and Reproduction, Maastricht University Medical Center+, Maastricht, The Netherlands; 2 Institute of Clinical Pathology and Molecular Pathology, Kepler University Hospital and Johannes Kepler University, Krankenhausstraße 9, 4021 Linz, Austria; 3 Pathology and Data Analytics, Leeds Institute of Medical Research at St. James’s, University of Leeds, Leeds, UK; 4 Department of Visceral Surgery and Medicine, Inselspital Bern, University of Bern, Bern, Switzerland; 5 Institute of Tissue Medicine and Pathology, University of Bern, Bern, Switzerland

**Keywords:** Gastric cancer, Metastases, PD-L1, Immunohistochemistry, Heterogeneity

## Abstract

**Background:**

Combination of immunotherapy and chemotherapy is recommended for first line treatment of gastric adenocarcinoma (GC) patients with locally advanced unresectable disease or metastatic disease. However, data regarding the concordance rate between PD-L1 combined positive score (CPS) in primary GC and matched regional lymph node metastasis (LNmet) or matched distant metastasis (Dmet) is limited.

**Methods:**

Tissue microarray sections from primary resected GC, LNmet and Dmet were immunohistochemically stained with anti-PD-L1 (clone SP263). PD-L1 expression was scored separately in tumour cells and immune cells and compared between matched primary GC, LNmet and/or Dmet. CPS was calculated and results for CPS cut-offs 1 and 5 were compared between matched samples.

**Results:**

275 PD-L1 stained GC were analysed. 189 primary GC had matched LNmet. CPS cut-off 1 concordance rate between primary GC and LNmet was 77%. 23 primary GC had matched Dmet but no matched LNmet, CPS cut-off 1 concordance rate was 70%. 63 primary GC had both matched LNmet and matched Dmet, CPS cut-off 1 concordance rate of 67%. CPS cut-off 5 results were similar. The proportion of PD-L1 positive tumour cells increased from primary GC (26%) to LNmet (42%) and was highest in Dmet (75%).

**Conclusion:**

Our study showed up to 33% discordance of PD-L1 CPS between primary GC and LNmet and/or Dmet suggesting that multiple biopsies of primary GC and metastatic sites might need to be tested before considering treatment options. Moreover, this is the first study that seems to suggest that tumour cells acquire PD-L1 expression during disease progression.

**Supplementary Information:**

The online version contains supplementary material available at 10.1007/s00432-023-05142-x.

## Background

Gastric cancer represents the fifth most common cancer worldwide and is one of the top 10 causes of cancer-related deaths in Europe (Ferlay et al. 2020). Programmed death-ligand 1 (PD-L1) / programmed cell death protein 1 (PD1) targeting immunotherapy is a promising new treatment option in locally advanced (unresectable) or metastatic gastric adenocarcinoma (GC). The combination of immunotherapy and chemotherapy is recommended as first line treatment for patients with locally advanced (unresectable) or metastatic gastric cancer according to the recent guidelines of the European Society for Medical Oncology (ESMO) (Lordick et al. 2022), the American National Comprehensive Cancer Network (NCCN) (Ajani et al. 2022) and the Japanese Gastric Cancer Association (JGCA) (Japanese Gastric Cancer Association 2023). The recommendation for the use of anti-PD1 monoclonal antibody nivolumab as immune checkpoint inhibitor treatment was mainly based on the results of the phase III CheckMate 649 trial (Janjigian et al. 2021a). As the response to immune checkpoint inhibition seems to be associated with higher expression levels of the PD-L1 protein in most tumour types, the PD-L1 status is nowadays routinely assessed by anti-PD-L1 immunohistochemistry in tumour tissue and semi-quantitatively reported as combined positive score (CPS) (Kulangara et al. 2019). At least 100 tumour cells need to be present in the PD-L1 stained tissue section to allow calculation of the CPS (total number of tumour cells, lymphocytes and macrophages with PD-L1 expression divided by the total number of tumour cells, multiplied by 100).

Many different PD-L1 assays and cut-offs (CPS 1, 5 or 10) were analysed in clinical trials (Janjigian et al. 2021a; Tabernero et al. 2021; Chung et al. 2021; Janjigian et al. 2021b). Previously published studies have compared the proportion of positive cases using different PD-L1 assays. Two different PD-L1 assays, based on the antibody clones 22C3 and SP263, showed similar proportions of PD-L1 positive cases for CPS ≥ 1 (58% versus 61% for 22C3 and SP263, respectively) in patients with stage II and III GC (Park et al. 2020). However, another study suggested that the use of the antibody clone 28–8 results in approximately two-fold higher proportion of PD-L1 positive GC than the use of the antibody clone 22C3 in different CPS cut-off scenarios (1, 5 or 10) (Yeong et al. 2022). Furthermore, the well-known heterogeneity of GC may affect the results. Our own previous study highlighted the intratumoural spatial heterogeneity between the primary resected GC and matched regional lymph node metastases (Sundar and Liu et al. 2021). One study analysed PD-L1 expression (antibody clone E1L3N) in 465 treatment naïve primary tumours and 15 resected liver metastases from patients with gastric or gastroesophageal junction cancer (Boger et al. 2016). PD-L1 expression was scored separately in tumour cells and immune cells. The authors reported that PD-L1 expression in tumour cells was seen in 30% (140/465) of the primary GC whereas 88% (411/465) of the primary GC showed PD-L1 expression in immune cells. Concordant PD-L1 expression in both, tumour cells and immune cells, was seen in 80% of matched primary GC and liver metastases. A recent retrospective study analysing PD-L1 expression (antibody clone 22C3) using CPS cut-off 1 reported a slightly lower concordance rate of 61% between 407 primary and metastatic tumour samples from 189 stage II-IV gastroesophageal cancer patients (Zhou et al. 2020).

Data regarding the concordance of PD-L1 expression in primary GC and matched regional lymph node metastasis or distant metastasis are limited. Most studies were performed before the publication of recent ESMO, NCCN and JGCA guidelines using scoring systems other than CPS and different PD-L1 antibodies. Inconsistent use of scoring systems and CPS cut-offs can be easily found in the clinical trials (Janjigian et al. 2021a; Tabernero et al. 2021; Chung et al. 2021; Janjigian et al. 2021b). In lung cancer, different PD-L1 assays (SP263, 22C3, 28–8, SP142, and 73–10) were tested in tumour samples in the Blueprint Phase 2 Project (Tsao et al. 2018). Comparable results were observed using these three PD-L1 assays SP263, 22C3 and 28.8. Other studies analysed the PD-L1 expression between primary tumour and metastases. For example, the PD-L1 expression was shown to be concordant between primary tumour and lymph node metastases in 62% of lung adenocarcinoma cases (Uruga et al. 2017).

Although PD-L1 expression with different assays has been investigated in GC (Janjigian et al. 2021a; Tabernero et al. 2021; Chung et al. 2021; Janjigian et al. 2021b), PD-L1 expression using CPS in treatment naïve primary GC and matched regional lymph node metastasis and/or distant metastasis has not been investigated in detail. PD-L1 expression data separately scored for tumour cells and immune cells in all three tumour locations are lacking. Therefore, the aim of our study was to investigate PD-L1 expression in tumour cells and immune cells separately using immunohistochemistry and compare the PD-L1 combined positive score (CPS) between the primary GC and matched regional lymph node metastasis and/or distant metastasis using CPS cut-off 1 and 5.

## Material and methods

### Study material

A total of 418 patients with gastric adenocarcinoma (GC) treated with primary surgery (without neoadjuvant treatment) between 1993 and 2013 at the Department of Surgery, Inselspital Bern, University of Bern, Switzerland was included in the study irrespective of the presence or absence of regional lymph node metastases in the resection specimen. Patients with gastric stump cancer or gastroesophageal junction cancer were excluded. Biopsies from distant metastases were obtained at the time of the initial diagnosis or during follow up. Detailed clinicopathological characteristics of the study cohort have been published in a previous study (Dislich et al. 2020). Tissue microarrays were constructed previously (core size 0.6 mm) from the primary GC, lymph node metastases and distant metastases. PD-L1 stained tissue microarray sections of 275 GC patients were eligible for the analyses. Approval for the study was given by the local ethical committee (University of Bern, Switzerland, no. 200/14).

### Immunohistochemistry and in situ hybridisation

Mismatch-repair (MMR) proteins MLH1, MSH2, MSH6 and PMS2 immunohistochemistry, EBER in situ hybridisation (Bond ready-to-use probe Leica Biosystems) and PD-L1 (clone SP263, Ventana Medical Systems) immunohistochemistry were performed as part of previous studies (Dislich et al. 2020, 2022).

For the purpose of the current study, the PD-L1 expression data from the previous study were used (Dislich 2022). Two CPS cut-offs were established in the current study and cases were regarded as PD-L1 positive with CPS ≥ 1 or CPS ≥ 5. Additionally, the PD-L1 expression of the tumour cells and the immune cells was scored separately. The proportion of tumour cells with PD-L1 expression was scored into five groups: 0% (no tumour cells with PD-L1 expression), less than 1% (i.e., > 0 to < 1), 1% to less than 5% (i.e., ≥ 1 to < 5), 5% to less than 50% (i.e., ≥ 5 to < 50), and 50% and more (i.e., ≥ 50). The same scoring system was used for scoring the immune cells.

### Statistical analyses

The relationship between the combined positive score (CPS < 1 versus CPS ≥ 1; CPS < 5 versus CPS ≥ 5) in two different tumour locations was analysed with the Chi-Square test. The P-values of the Pearson Chi-Square or Fisher’s exact test are reported. The two-sided test with P-value < 0.05 was regarded as statistically significant. All statistical analyses were performed with SPSS 27.0 (IBM, Somers, NY, USA).

## Results

For this programmed death-ligand 1 (PD-L1) study, stained tissue microarray sections of 275 gastric cancer (GC) patients were eligible for the analyses. This cohort consisted of 189 (68.7%) primary GC with matched regional lymph node metastases only, 23 (8.4%) primary GC with matched distant metastasis only, and 63 (22.9%) primary GC with both, matched regional lymph node metastasis and distant metastasis. The median age of the patients was 71 years (range: 31 to 92 years). 63% of patients were male. 87% of patients were classified as having pT3 or pT4 GC. The clinicopathological characteristics of this cohort are summarized in Online Resource 1.

### Primary gastric adenocarcinoma with matched regional lymph node metastasis without distant metastasis

#### Combined positive score (CPS) cut-off 1

**Fig. 1:**
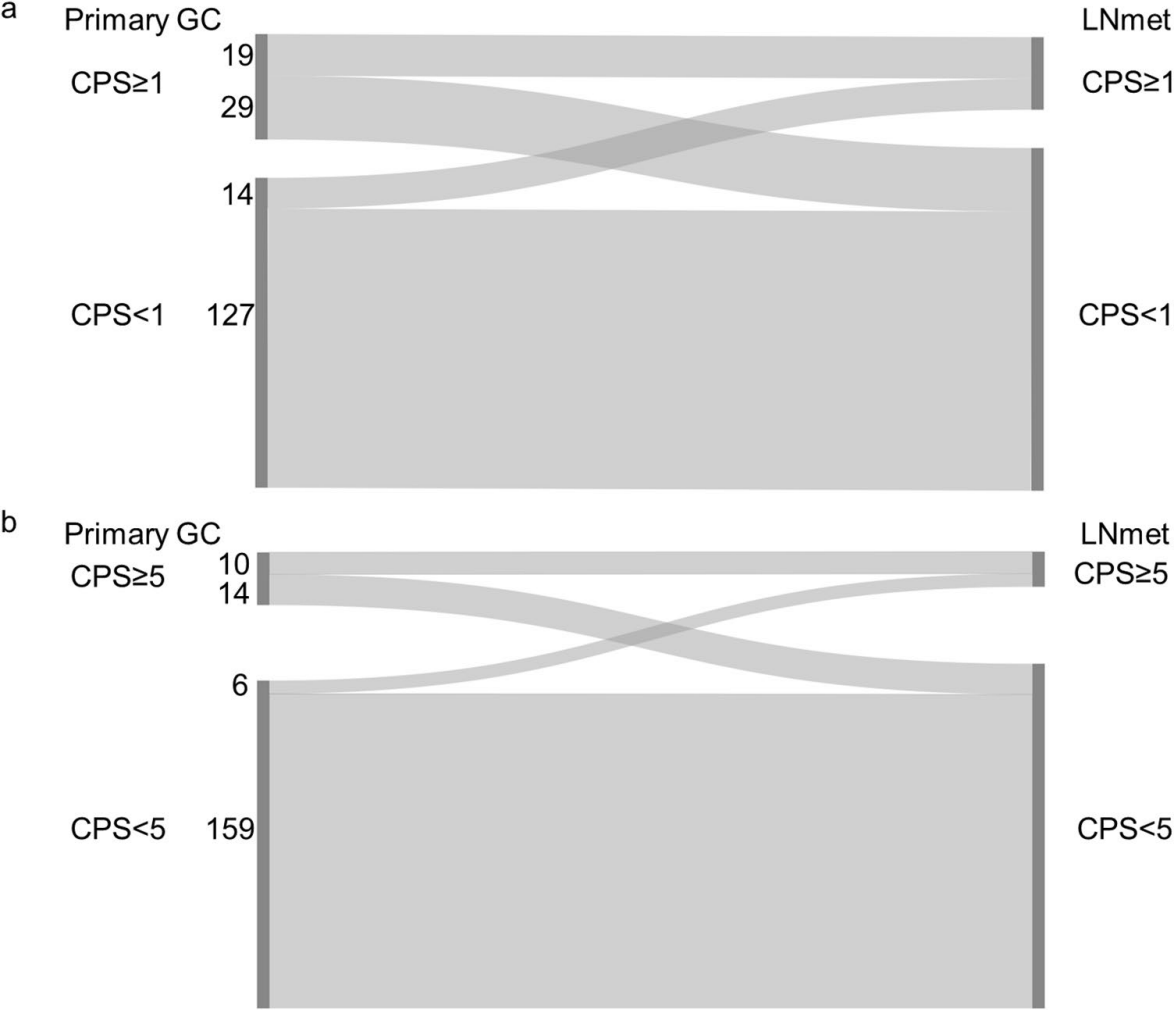
Combined positive score (CPS) in matched primary gastric adenocarcinoma (GC) and lymph node metastasis (LNmet) (n = 189) **a** Sankey diagram showing the change in CPS between matched samples scored using CPS cut-off 1. The left side of the diagram shows the number of primary GC cases with CPS ≥ 1 or CPS < 1. The right side of the diagram shows the number of matched LNmet with CPS ≥ 1 or CPS < 1. Ribbons connect matched samples. **b** Sankey diagram showing the change in CPS between matched cases scored using CPS cut-off 5. The left side of the diagram shows the number of primary GC cases with CPS ≥ 5 or CPS < 5. The right side of the diagram shows the number of matched LNmet with CPS ≥ 5 or CPS < 5. Ribbons connect matched samples

25.4% (48/189) of the primary GC and 17.5% (33/189) of the matched regional lymph node metastases showed CPS ≥ 1. The CPS concordance rate between the primary GC and the matched regional lymph node metastasis was 77.2% (Fig. 1a and Online Resource 2).

#### Combined positive score (CPS) cut-off 5

12.7% (24/189) of the primary GC and 8.5% (16/189) of the matched regional lymph node metastases showed CPS ≥ 5. The CPS concordance rate between the primary GC and the matched regional lymph node metastasis was 89.4% (Fig. 1b and Online Resource 2).

### Primary gastric adenocarcinoma with matched distant metastasis without regional lymph node metastasis

#### Combined positive score (CPS) cut-off 1

**Fig. 2:**
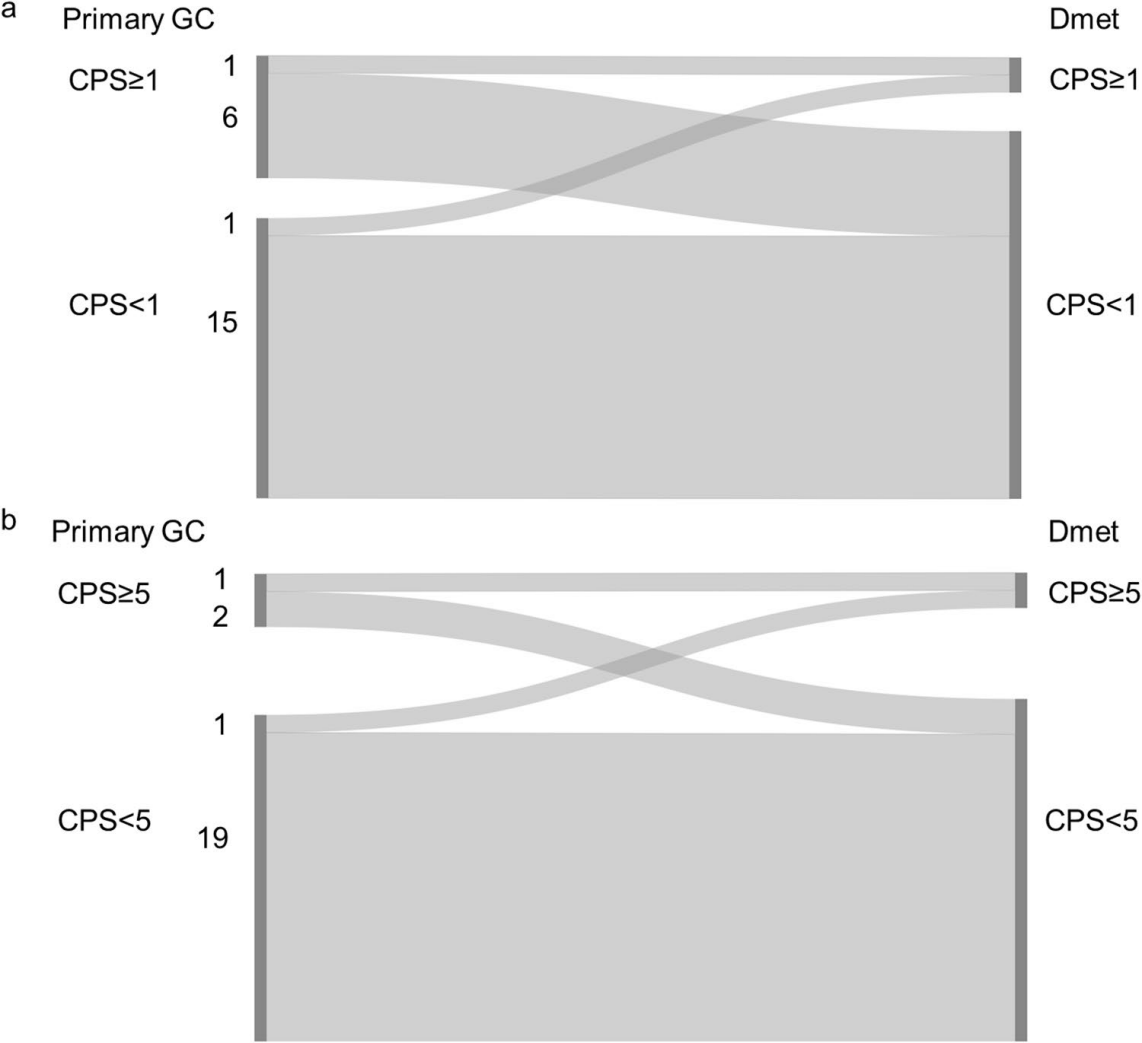
Combined positive score (CPS) in matched primary gastric adenocarcinoma (GC) and distant metastasis (Dmet) (n = 23), **a** Sankey diagram showing the change in CPS between matched samples scored using CPS cut-off 1. The left side of the diagram shows the number of primary GC cases with CPS ≥ 1 or CPS < 1. The right side of the diagram shows the number of matched Dmet with CPS ≥ 1 or CPS < 1. Ribbons connect matched samples. **b** Sankey diagram showing the change in CPS between matched samples scored using CPS cut-off 5. The left side of the diagram shows the number of primary GC cases with CPS ≥ 5 or CPS < 5. The right side of the diagram shows the number of matched Dmet with CPS ≥ 5 or CPS < 5. Ribbons connect matched samples

30.4% (7/23) of the primary GC and 8.7% (2/23) of the matched distant metastases showed CPS ≥ 1. The CPS concordance rate between the primary GC and the matched distant metastasis was 69.6% (Fig. 2a and Online Resource 3).

#### Combined positive score (CPS) cut-off 5

13.0% (3/23) of the primary GC and 8.7% (2/23) of the matched distant metastases showed CPS ≥ 5. The CPS concordance rate between the primary GC and the matched distant metastases was 87.0% (Fig. 2b and Online Resource 3).

### Primary gastric adenocarcinoma with matched regional lymph node metastasis and matched distant metastasis

#### Combined positive score (CPS) cut-off 1

63.5% (40/63) patients had a CPS < 1 in the primary GC and in both metastatic locations. Only 3.2% (2/63) patients had a CPS ≥ 1 in all three locations (primary GC, lymph node metastasis and distant metastasis). The CPS concordance rate between the primary GC, matched regional lymph node metastasis and matched distant metastasis was 66.7%.

**Fig. 3:**
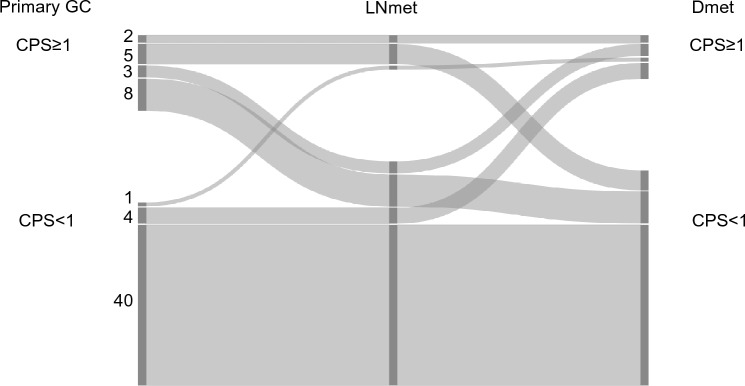
Combined positive score (CPS) cut-off 1 in matched primary gastric adenocarcinoma (GC), lymph node metastasis (LNmet) and distant metastasis (Dmet) (n = 63). Sankey plot showing the change in CPS between matched samples scored using CPS cut-off 1. The left side of the diagram shows the number of primary GC cases with CPS ≥ 1 or CPS < 1. The right side of the diagram shows the number of matched LNmet and Dmet with CPS ≥ 1 or CPS < 1. Ribbons connect matched samples

In the remaining 33.3% (21/63) patients, CPS was different in at least one of the three matched tumour locations, see Fig. 3.

#### Combined positive score (CPS) cut-off 5

69.8% (44/63) patients had a CPS < 1 in the primary GC and in both metastatic locations. Only 1.6% (1/63) patients had a CPS ≥ 1 in all three locations (primary GC, lymph node metastasis and distant metastasis). The CPS concordance rate between the primary GA and both matched regional lymph node metastasis and matched distant metastasis was 71.4%.

**Fig. 4:**
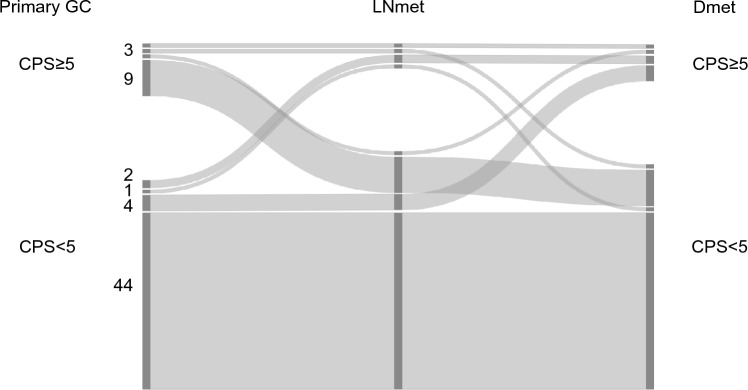
Combined positive score (CPS) cut-off 5 in matched primary gastric adenocarcinoma (GC) and lymph node metastasis (LNmet) and distant metastasis (Dmet) (n = 63). Sankey diagram showing the change in CPS between matched samples scored using CPS cut-off 5. The left side of the diagram shows the number of primary GC cases with CPS ≥ 5 or CPS < 5. The right side of the diagram shows the number of matched LNmet and Dmet with CPS ≥ 5 or CPS < 5. Ribbons connect matched samples

In the remaining 28.6% (18/63) patients, CPS was different in at least one of the three matched tumour locations, see Fig. 4.

### Heterogeneity of PD-L1 expression in tumour cells and immune cells in different tumour locations

#### Primary gastric adenocarcinoma

73 (26.5%) primary GC had a CPS ≥ 1. In 54 (74%) of primary GC the CPS ≥ 1 score was solely due to PD-L1 expression in immune cells and tumour cells showed no PD-L1 expression at all (category 0 in PD-L1 expression; Table 1).

**Table 1:** Frequencies of programmed death-ligand 1 (PD-L1) SP263 expression in tumour cells versus immune cells of combined positive score (CPS) ≥ 1 in primary gastric adenocarcinoma (GC), lymph node metastases and distant metastases

	Proportion cells with PD-L1 expression n (%)				
	0*	> 0 to < 1	≥ 1 to < 5	≥ 5 to < 50	≥ 50
Primary GC n = 73					
Tumour cells	54 (74.0)	2 (2.7)	5 (6.8)	8 (11.0)	4 (5.5)
Immune cells	0 (0.0)	0 (0.0)	45 (61.6)	27 (37.0)	1 (1.4)
Lymph node metastases n = 41					
Tumour cells	24 (58.5)	1 (2.4)	3 (7.3)	4 (9.8)	9 (22.0)
Immune cells	3 (7.3)	0 (0.0)	25 (61.0)	13 (31.7)	0 (0.0)
Distant metastases n = 12					
Tumour cells	3 (25.0)	2 (16.7)	0 (0.0)	5 (41.7)	2 (16.7)
Immune cells	1 (8.3)	0 (0.0)	6 (50.0)	5 (41.7)	0 (0.0)

*PD-L1 expression was scored into 5 groups: 0% (no tumour cells or immune cells with PD-L1 expression), less than 1% (i.e., > 0 to < 1), 1% to less than 5% (i.e., ≥ 1 to < 5), 5% to less than 50% (i.e., ≥ 5 to < 50), and 50% and more (i.e., ≥ 50)

#### Lymph node metastases

41 (14.9%) lymph node metastases had a CPS ≥ 1. 24 (58.5%) lymph node metastases did not show PD-L1 expression in the tumour cells (category 0 in PD-L1 expression; Table 1), whereas the immune cells showed PD-L1 expression (categories ≥ 1 to < 50 in PD-L1 expression) in 38 (92.7%) of lymph node metastases.

#### Distant metastases

There were 12 (4.4%) distant metastases with CPS ≥ 1. The PD-L1 expression in tumour cells in the distant metastases was heterogeneous. Five cases showed no PD-L1 expression (categories 0 and > 0 to < 1; Table 1) in tumour cells. It appears that the distant metastases with CPS ≥ 1 were due to the positive PD-L1 expression in the immune cells (11/12; categories ≥ 1 to < 50).

As PD-L1 expression was scored separately in tumour cells and immune cells, we were able to analyse and compare which cell type contributed most to a CPS ≥ 1 in the primary GC, matched regional lymph node metastasis or matched distant metastasis. In patients where the GC was scored as CPS ≥ 1 in all three tumour locations, 74.0% of primary GC, 58.8% of lymph node metastases and 25.0% of distant metastases showed no PD-L1 expression in tumour cells (Table 1). Thus, the proportion of tumour cells with PD-L1 expression increased from primary GC to lymph node metastases and was highest in distant metastases.

Similar results were found for CPS cut-off 5 (Online Resource 4). Examples of concordant and heterogenous PD-L1 expression of primary GC with both matched lymph node metastasis and distant metastasis from three patients can be found in Online Resource 5.

## Discussion

PD-L1 expression has not been investigated in detail in chemotherapy naïve resected matched primary gastric adenocarcinoma (GC), regional lymph node metastasis and/or distant metastasis. Similarly, data on PD-L1 expression of tumour cells and immune cells separate in all three sites are limited. Thus, we investigated PD-L1 expression in tumour cells and immune cells separately using immunohistochemistry and compared the PD-L1 combined positive score (CPS) with cut-off 1 and 5 between the three sites. The cohort consisted of 189 primary GC with matched regional lymph node metastasis only, 23 primary GC with matched distant metastasis only, and 63 primary GC with both matched lymph node metastasis and distant metastasis. Results were similar for CPS cut-off 1 and 5.

The majority of the primary GC in the current study showed CPS < 1 or CPS < 5. This is in contrast to most of the previous studies. 62% of Japanese advanced GC had a PD-L1 expression of CPS ≥ 1 (Yoshida et al. 2022). Similarly, 67% (16/24 patients of cohort 2) of advanced gastric or gastroesophageal junction cancers from the phase II nonrandomized KEYNOTE-059 study had a PD-L1 expression of CPS ≥ 1 (Bang et al. 2019). When CPS ≥ 10 was used as a cut-off, the prevalence of PD-L1 positive cancers was 37% (281/763 patients) in the randomized KEYNOTE-062 study of advanced gastric or gastroesophageal junction cancers (Chao et al. 2021) and 18% (108/592 patients) in the randomized phase 3 KEYNOTE-061 trial (Fuchs et al. 2022). 67% (395/592 patients) had CPS ≥ 1 in this trial (Fuchs et al. 2022). However, gastroesophageal junction adenocarcinomas were included in these previous studies, whereas the current study focussed on gastric cancers. The use of different PD-L1 assays, different cut-offs for definition of positive PD-L1 status and different scoring systems (tumour proportion score versus CPS) makes direct comparison of our results with previous results difficult.

The CPS cut-off 1 concordance rate in the current study was minimal 66.7% between the primary GC and metastases which is similar to a previous smaller study in 62 patients with gastroesophageal cancers by Zhou et al. (Zhou et al. 2020). Findings in GC, seem to be similar to those reported in lung cancer (Uruga et al. 2017; Takamori et al. 2017; Mansfield et al. 2016), whereas the concordance rate of PD-L1 expression was much higher comparing primary colorectal cancer and brain metastases based on the PD-L1 expression in only the tumour cells (≥ 1% of tumour cells with membranous staining was considered positive) (Roussille et al. 2018).

Our analyses comparing PD-L1 expression of the primary GC with matched regional lymph node metastasis and distant metastasis suggests that the proportion of tumour cells with PD-L1 expression increases with tumour progression being lowest in the primary GC and highest in the distant metastasis. Thus, multiple biopsies of primary GC and/or metastatic sites might need to be tested before considering treatment options. In other cancer types, such as in clear cell renal cell cancers, a higher proportion of PD-L1 expression was observed in the matched lung or lymph node metastases (33%; 27/83 patients) than in the primary tumour (24%; 20/83 patients). However, this study used a cut-off of 5% and only scored tumour cells with PD-L1 expression (clone ZM-0170) (Zhang et al. 2019).

Our study has several limitations. This is a retrospective study with a relatively small number of patients with matched lymph node metastasis and distant metastasis and hence, the results comparing all three locations need to be interpreted with caution. Nevertheless, this is currently the largest study comparing 275 patients with matched primary GC and lymph node metastasis and/or distant metastasis, which did not receive neoadjuvant chemotherapy before surgery of the primary GC. All patients were treated in a single institution, which may have introduced selection bias. Analyses of the tumour samples were performed using tissue microarrays and metastases (targeted regions due to limited tumour tissues). The current study investigated tumour samples from patients with resectable disease. Thus, it is unknown whether results would be similar in patients with stage III or IV disease.

## Conclusion

This is the largest study investigating matched primary gastric adenocarcinoma (GC) and lymph node metastasis and/or distant metastasis from 275 patients without neoadjuvant chemotherapy. Our study focused on the programmed death-ligand 1 (PD-L1) expression in matched tumour samples and analysed separately PD-L1 expression in tumour cells and immune cells in these three tumour locations by immunohistochemistry using the combined positive score (CPS). PD-L1 expression was heterogeneous in matched primary GC and metastases with a CPS discordance rate up to 33% in the current study. In contrast to previous studies, the prevalence of GC with PD-L1 expression was less than 30%. Interestingly, it is likely that the proportion of tumour cells with PD-L1 expression increases from the primary GC to the metastatic sites, suggesting that the tumour cells acquire PD-L1 expression during disease progression. Thus, multiple biopsies of primary GC and metastatic sites might need to be tested before considering treatment options. Further research is needed to elucidate the biological mechanism of the changing PD-L1 expression of the tumour cells and the immune cells at different tumour sites, as well as PD-L1 expression before and after chemotherapy treatment.

### Supplementary Information

Below is the link to the electronic supplementary material.

Supplementary file1 (DOCX 15 KB)

Supplementary file2 (DOCX 17 KB)

Supplementary file3 (DOCX 17 KB)

Supplementary file4 (DOCX 18 KB)

Supplementary file5 (PPTX 1139 KB)

## Data Availability

The data used to support the findings of this study are available from the corresponding author upon reasonable request.
