# Supplementary material for: Programmed death-ligand 1 (PD-L1) expression in primary gastric adenocarcinoma and matched metastases
Source: J Cancer Res Clin Oncol. 2023 Jul 25;149(14):13345–52. doi: 10.1007/s00432-023-05142-x (PMC10587283; doi:10.1007/s00432-023-05142-x)
Supplement: Supplementary file 1 — Supplementary file1 (DOCX 15 KB) [file 432_2023_5142_MOESM1_ESM.docx]

Online Resource 1 Clinicopathological characteristics of patients included in the programmed death-ligand 1 cohort (n = 275)

|  | Total number  n (%) |
| --- | --- |
| Sex |  |
| Male | 174 (63.3) |
| Female | 101 (36.7) |
| pT category |  |
| T1 | 9 (3.3) |
| T2 | 26 (9.5) |
| T3 | 106 (38.5) |
| T4 | 134 (48.7) |
| pN category* |  |
| N0 | 10 (3.6) |
| N1-3 | 264 (96.0) |
| pM category |  |
| M0 | 179 (65.1) |
| M1 | 96 (34.9) |
| Laurén classification* |  |
| Intestinal | 126 (45.8) |
| Diffuse | 98 (35.6) |
| Mixed | 47 (17.1) |
| Mismatch repair* |  |
| Deficient | 21 (7.6) |
| Proficient | 252 (91.6) |
| Epstein-Barr virus |  |
| Positive | 8 (2.9) |
| Negative | 267 (97.1) |

*The percentages do not add up to 100%, because of the missing data.

pTNM stage according to 8^th^ edition of UICC TNM Classification
