# Supplementary material for: Programmed death-ligand 1 (PD-L1) expression in primary gastric adenocarcinoma and matched metastases
Source: J Cancer Res Clin Oncol. 2023 Jul 25;149(14):13345–52. doi: 10.1007/s00432-023-05142-x (PMC10587283; doi:10.1007/s00432-023-05142-x)
Supplement: Supplementary file 2 — Supplementary file2 (DOCX 17 KB) [file 432_2023_5142_MOESM2_ESM.docx]

Online Resource 2 Two-by-two contingency tables of the combined positive score (CPS) cut-offs between primary gastric adenocarcinoma (GC) and matched lymph node metastasis (n = 189)

|  | Lymph node metastasis | |  |  |
| --- | --- | --- | --- | --- |
|  | CPS < 1  n (%) | CPS ≥ 1  n (%) | Total number | P-value |
| Primary GC  CPS < 1  CPS ≥ 1 | 127 (90.1)  29 (60.4) | 14 (9.9)  19 (39.6) | 141  48 | < 0.001 |
|  | **Lymph node metastasis** | |  |  |
|  | CPS < 5  n (%) | CPS ≥ 5  n (%) |  |  |
| Primary GC  CPS < 5  CPS ≥ 5 | 159 (96.4)  14 (58.3) | 6 (3.6)  10 (41.7) | 165  24 | < 0.001 |
