# Supplementary material for: Programmed death-ligand 1 (PD-L1) expression in primary gastric adenocarcinoma and matched metastases
Source: J Cancer Res Clin Oncol. 2023 Jul 25;149(14):13345–52. doi: 10.1007/s00432-023-05142-x (PMC10587283; doi:10.1007/s00432-023-05142-x)
Supplement: Supplementary file 3 — Supplementary file3 (DOCX 17 KB) [file 432_2023_5142_MOESM3_ESM.docx]

Online Resource 3 Two-by-two contingency tables of the combined positive score (CPS) cut-offs between primary gastric adenocarcinoma (GC) and matched distant metastasis (n = 23)

|  | Distant metastasis | |  |  |
| --- | --- | --- | --- | --- |
|  | CPS < 1  n (%) | CPS ≥ 1  n (%) | Total number | P-value |
| Primary GC  CPS < 1  CPS ≥ 1 | 15 (93.8)  6 (85.7) | 1 (6.3)  1 (14.3) | 16  7 | 0.526 |
|  | **Distant metastasis** | |  |  |
|  | CPS < 5  n (%) | CPS ≥ 5  n (%) |  |  |
| Primary GC  CPS < 5  CPS ≥ 5 | 19 (95.0)  2 (66.7) | 1 (5.0)  1 (33.3) | 20  3 | 0.249 |
