# Supplementary material for: Programmed death-ligand 1 (PD-L1) expression in primary gastric adenocarcinoma and matched metastases
Source: J Cancer Res Clin Oncol. 2023 Jul 25;149(14):13345–52. doi: 10.1007/s00432-023-05142-x (PMC10587283; doi:10.1007/s00432-023-05142-x)
Supplement: Supplementary file 4 — Supplementary file4 (DOCX 18 KB) [file 432_2023_5142_MOESM4_ESM.docx]

Online Resource 4 Frequencies of programmed death-ligand 1 (PD-L1) SP263 expression in tumour cells versus immune cells of combined positive score (CPS) ≥ 5 in primary gastric adenocarcinoma (GC), lymph node metastases and distant metastases

|  | Proportion cells with PD-L1 expression  n (%) | | | | |
| --- | --- | --- | --- | --- | --- |
|  | 0* | > 0 to < 1 | ≥ 1 to < 5 | ≥ 5 to < 50 | ≥ 50 |
| Primary GC  n = 39 |  |  |  |  |  |
| Tumour cells | 20  (51.3) | 2  (5.1) | 5  (12.8) | 8  (20.5) | 4  (10.3) |
| Immune cells | 0  (0.0) | 0  (0.0) | 11  (28.2) | 27  (69.2) | 1  (2.6) |
|  |  |  |  |  |  |
| Lymph node metastases  n = 21 |  |  |  |  |  |
| Tumour cells | 5  (23.8) | 1  (4.8) | 2  (9.5) | 4  (19.0) | 9  (42.9) |
| Immune cells | 2  (9.5) | 0  (0.0) | 6  (28.6) | 13  (61.9) | 0  (0.0) |
|  |  |  |  |  |  |
| Distant metastases  n = 10 |  |  |  |  |  |
| Tumour cells | 1  (10.0) | 2  (20.0) | 0  (0.0) | 5  (50.0) | 2  (20.0) |
| Immune cells | 1  (10.0) | 0  (0.0) | 4  (40.0) | 5  (50.0) | 0  (0.0) |

*PD-L1 expression was scored into 5 groups: 0% (no tumour cells or immune cells with PD-L1 expression), less than 1% (i.e., > 0 to < 1), 1% to less than 5% (i.e., ≥ 1 to < 5), 5% to less than 50% (i.e., ≥ 5 to < 50), and 50% and more (i.e., ≥ 50)
