# Supplementary material for: Programmed death-ligand 1 (PD-L1) expression in primary gastric adenocarcinoma and matched metastases
Source: J Cancer Res Clin Oncol. 2023 Jul 25;149(14):13345–52. doi: 10.1007/s00432-023-05142-x (PMC10587283; doi:10.1007/s00432-023-05142-x)
Supplement: Supplementary file 5 — Supplementary file5 (PPTX 1139 KB) [file 432_2023_5142_MOESM5_ESM.pptx]

## Slide 1
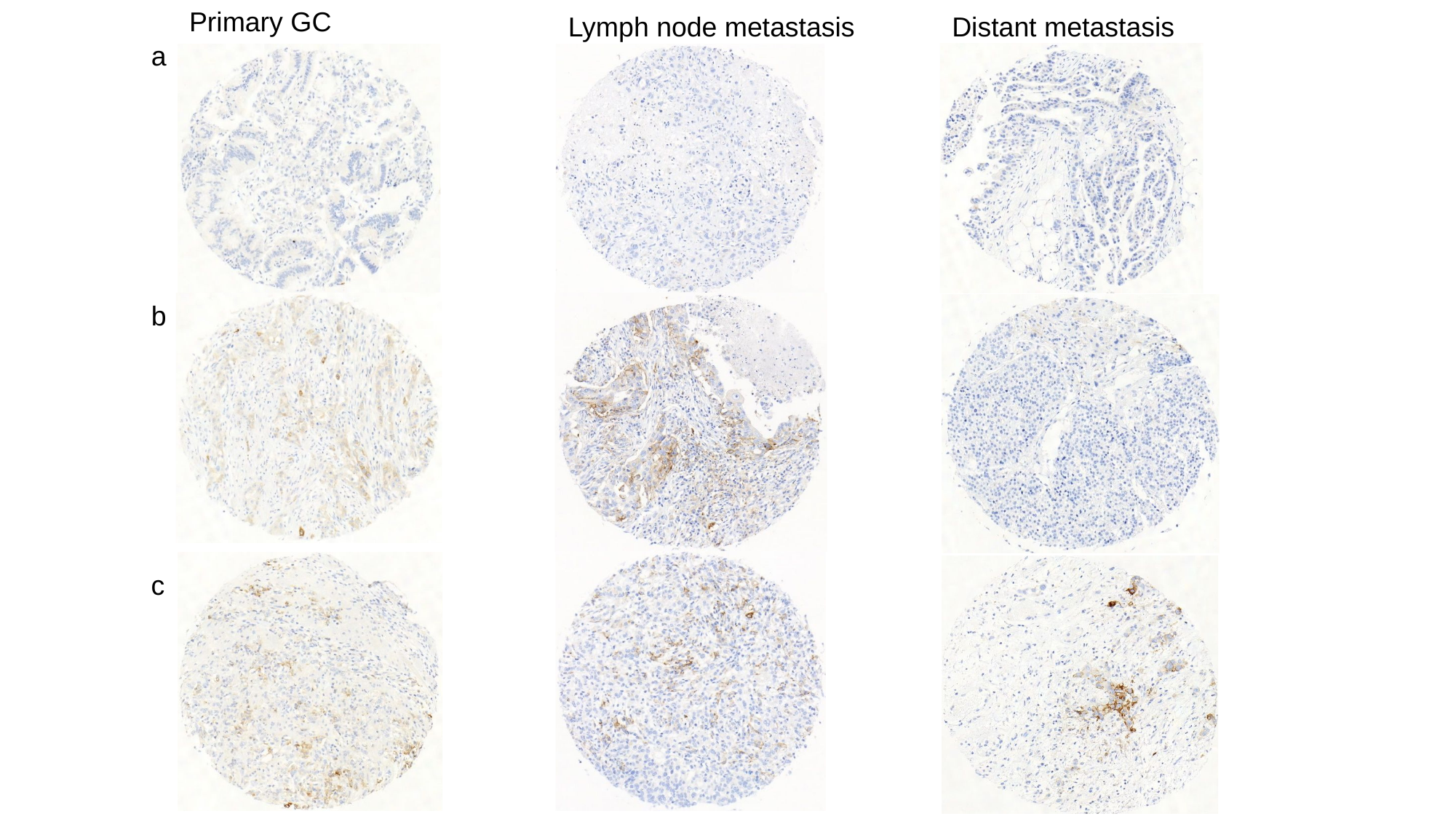

Primary GC
Lymph node metastasis
Distant metastasis
a
b
c

## Slide 2
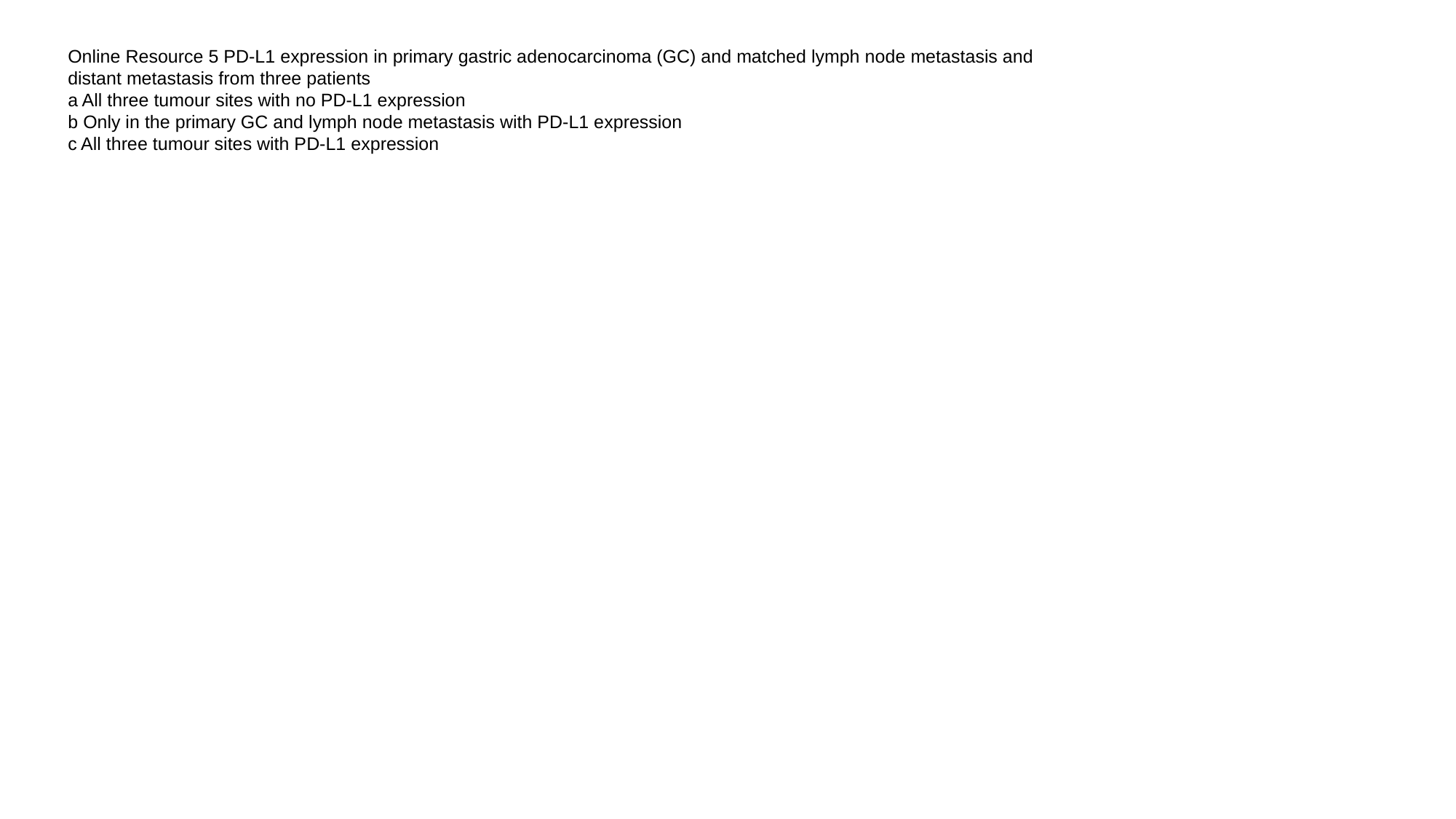

Online Resource 5 PD-L1 expression in primary gastric adenocarcinoma (GC) and matched lymph node metastasis and distant metastasis from three patients
a All three tumour sites with no PD-L1 expression
b Only in the primary GC and lymph node metastasis with PD-L1 expression
c All three tumour sites with PD-L1 expression
